# 65 & Thrive: Improving Patient Length of Stay, Readmission, and Quality of Care by Becoming an Age-Friendly Hospital

**DOI:** 10.1093/geroni/igab046.2278

**Published:** 2021-12-17

**Authors:** Tracey Vien, Stella Bobroff, Ricardo De Ocampo

**Affiliations:** 1 Kaiser Permanente Med Group/Southern California, Los Angeles, California, United States; 2 Kaiser Permanente, Los Angeles, California, United States

## Abstract

Data indicates that older persons will increase in numbers along with having an increase of life expectancy in the United States. Kaiser Permanente Los Angeles Medical Center’s Utilization Department developed “65 & Thrive”—an age-specialized initiative to provide holistic care that preserves independence, quality of life, prevents functional and cognitive decline, and promotes both patients and their families to continue thriving. The initiative’s focus is guided by the 5 M’s model on mobility, medication, mentation, multi-morbidity, and what matters. Case management staff were given age-sensitivity trainings, improved workflows and made assessments that identified, addressed, and secured resources for patients throughout their hospitalization. Silver Angel volunteers were specially trained to prevent physical and mental decline and focused on activities to prevent delirium, depression and falls. The volunteers visited with patients daily for these interactions. The initiative was piloted in April 2020 on a stroke telemetry unit and since then the hospital has seen a significant decrease in the overall annual readmission rates by 3.1% when compared to 2019. The average length of stay for older adult patients; however, increased from 4.05 to 4.83 days unfortunately due to COVID-19. This initiative demonstrates the necessity to expand “65 & Thrive” throughout the hospital and ultimately to other Kaiser Permanente medical centers to best provide holistic care to older adults.

